# Unusual presentation of Lisfranc fracture dislocation associated with high-velocity sledding injury: a case report and review of the literature

**DOI:** 10.1186/1752-1947-2-266

**Published:** 2008-08-11

**Authors:** Christopher E Benejam, Steven G Potaczek

**Affiliations:** 1 Augustana College, 38th Street, Rock Island, IL, 61201, USA; 2 Department of Orthopedic Surgery, Galesburg Clinic, N Seminary St, Galesburg, IL, 61401, USA

## Abstract

**Introduction:**

Lisfranc fracture dislocations of the foot are rare injuries. A recent literature search revealed no reported cases of injury to the tarsometatarsal (Lisfranc) joint associated with sledding.

**Case presentation:**

A 19-year-old male college student presented to the emergency department with a Lisfranc fracture dislocation of the foot as a result of a high-velocity sledding injury. The patient underwent an immediate open reduction and internal fixation.

**Conclusion:**

Lisfranc injuries are often caused by high-velocity, high-energy traumas. Careful examination and thorough testing are required to identify the injury properly. Computed tomography imaging is often recommended to aid in diagnosis. Treatment of severe cases may require immediate open reduction and internal fixation, especially if the risk of compartment syndrome is present, followed by a period of immobilization. Complete recovery may take up to 1 year.

## Introduction

An unusual case of Lisfranc fracture dislocation of the foot resulting from a high-velocity sledding injury is discussed. A recent literature search revealed no reported cases of injury to the tarsometatarsal (Lisfranc) joint associated with sledding.

## Case presentation

A healthy 19-year-old male college student presented to the emergency department with acute pain in the left foot after sustaining a sledding injury. While sledding in the sitting position and with legs extended, the plantar aspect of his left foot struck a tree limb at high speed. The pain was throbbing and did not radiate. Weight bearing was impossible. Previous medical and surgical records were unremarkable.

On physical examination, localized swelling and tenderness of the dorsal aspect of the midfoot prevented weight-bearing or movement of the foot and ankle. Circulation and neurological examinations were normal. The skin was intact.

**Figure 1:**
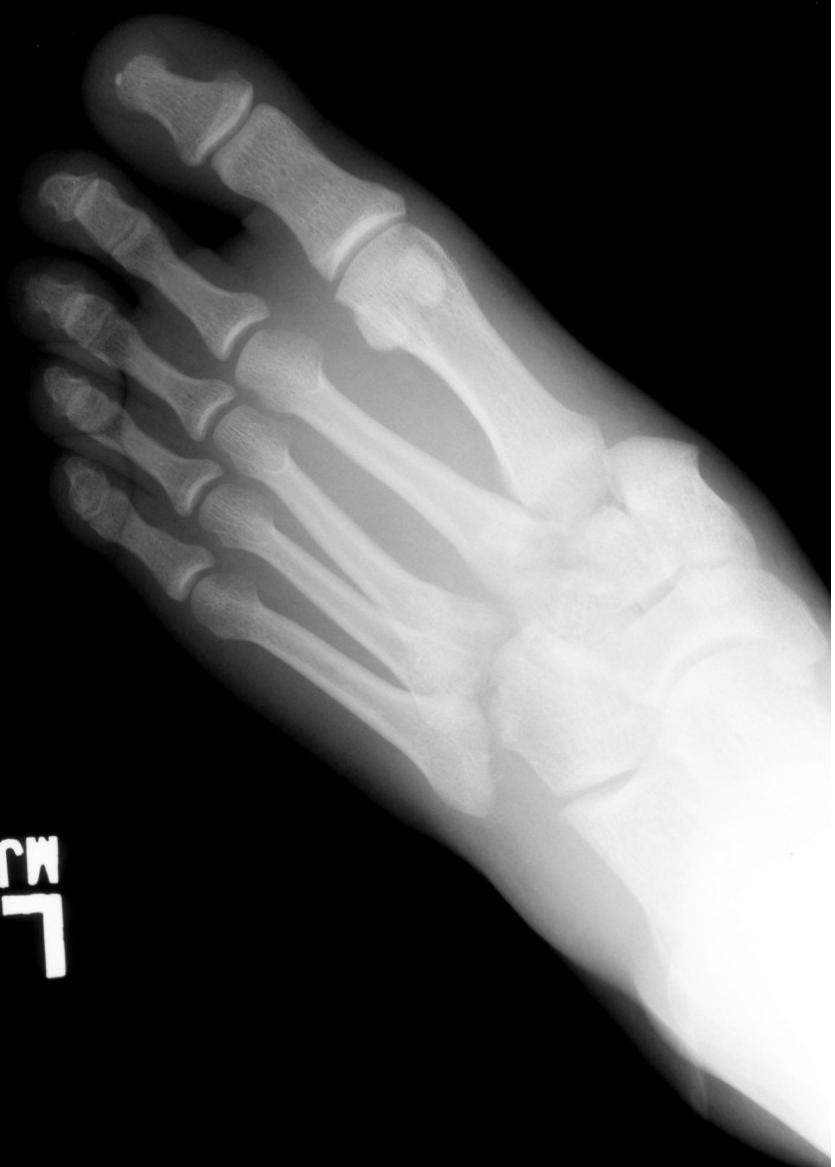
**Radiograph of the left foot.** There is lateral displacement of the first, second, and third metatarsals (tarsometatarsal or Lisfranc joint) with associated fracture of the middle cuneiform.

**Figure 2:**
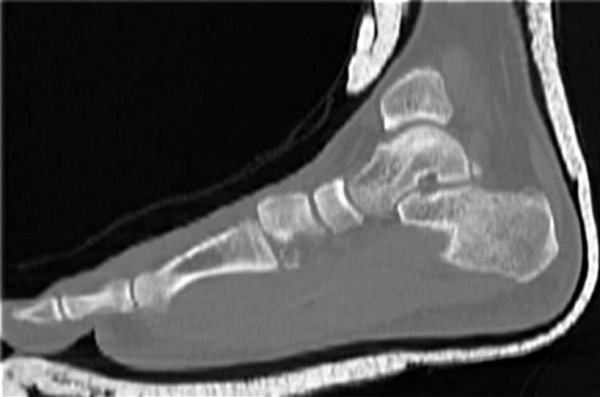
**Computed tomography of the left foot.** There is disruption of the tarsometatarsal (Lisfranc) joint with associated soft tissue swelling.

Foot radiograph demonstrated a Lisfranc fracture dislocation (Fig. 1). A subsequent CT scan is shown (Fig. 2).

**Figure 3:**
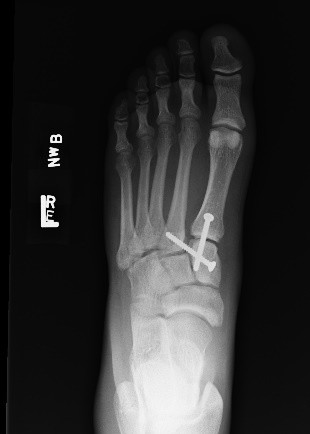
**Radiograph of the left foot. **There is anatomic alignment of the tarsometatarsal (Lisfranc) joint with a screw connecting the first metatarsal and the medial cuneiform, and a screw connecting the second metatarsal and the medial cuneiform.

This patient underwent an immediate open reduction and internal fixation of the Lisfranc fracture-dislocation. A postoperative radiograph is shown (Fig. 3). He was treated with a non-weight-bearing cast followed by a weight-bearing boot. He was advised to refrain from strenuous physical activity for 6 weeks after removal of the boot, after which time, normal physical activity was resumed. A non-steroidal anti-inflammatory drug was prescribed for pain. The patient had only mild pain with weight-bearing at 6 months and was ambulating without difficulty; he was pain-free at 2 years.

## Discussion

The Lisfranc joint derives its name from Jacques Lisfranc (1790–1847), a surgeon in Napoleon's army. Lisfranc performed amputations through the tarsometatarsal (TMT) joint to treat gangrenous injury of the foot [1]. Injuries of the Lisfranc joint are rare, representing less than 0.2% of all orthopedic traumas [2]. However, as many as 20% of Lisfranc joint injuries are missed upon initial examination [3]. The injury should always be suspected following trauma to the foot [4]. Most commonly, Lisfranc joint sprains and fractures are caused by high-velocity traumas, such as motor vehicle and industrial accidents. Injuries can be sustained during many athletic activities. In this case, injury was caused by direct impact of the foot against a tree trunk resulting in acute plantar flexion. In patients with high-energy trauma foot injury, CT imaging is often recommended to aid in diagnosis [5].

Mild sprains to the Lisfranc joint, where there is no evidence of diastasis, may be treated by immobilization [6]. Treatment of more severe cases such as dislocations, however, usually includes open reduction and internal fixation of the joint. Cortical screw fixation is preferred to Kirschner wire fixation for these injuries [7]. The joint is secured to reduce without diastasis the lateral border of the medial cuneiform to the second metatarsal [3]. Surgery may be postponed to allow for reduction in tissue edema. However, if a risk of compartment syndrome is present, surgery should be performed immediately. After surgery, the foot is immobilized in a non-weight-bearing cast for 6 to 8 weeks, after which, the foot may be placed in an immobilizing boot with minimal weight bearing. After an additional 6 to 8 weeks, the boot may be removed and full weight-bearing may be established gradually. Complete recovery often takes up to 1 year [3], although long-term disability is possible. Despite appropriate reduction and fixation, patients may develop chronic post-traumatic arthritis [8]. Primary complete arthrodesis as a salvage procedure [9] is recommended only for severe chronic pain.

## Conclusion

Lisfranc injuries are often caused by high-velocity traumas. Careful examination and thorough testing are required to identify the injury correctly, as a patient may present symptoms consistent with sprains or other minor injuries. Treatment of severe cases may require open reduction and internal fixation followed by a period of immobilization. Complete recovery may take up to 1 year.

## Consent

Written informed consent was obtained from the patient for publication of this case report and the accompanying images. A copy of the written consent is available for review by the Editor-in-Chief of this journal.

## Competing interests

The authors declare that they have no competing interests.

## Authors' contributions

CB wrote the first draft of the manuscript, obtained patient consent, and reviewed the literature. SP proofread the case report and provided revisions. All authors read and approved the final manuscript.
